# Additional Haptic Information Provided by Anchors Reduces Postural Sway in Young Adults Less Than Does Light Touch

**DOI:** 10.3389/fnins.2018.00346

**Published:** 2018-06-05

**Authors:** Renato Moraes, Bruno L. S. Bedo, Luciana O. Santos, Rosangela A. Batistela, Paulo R. P. Santiago, Eliane Mauerberg-deCastro

**Affiliations:** ^1^Laboratory of Biomechanics and Motor Control, School of Physical Education and Sport of Ribeirão Preto, University of São Paulo, Ribeirão Preto, Brazil; ^2^Action and Perception Laboratory, Department of Physical Education, São Paulo State University, Rio Claro, Brazil

**Keywords:** postural control, balance, haptic cue, foot position, stability

## Abstract

This study investigated the effect of adding haptic information to the control of posture, as well as comparing the effect of both the “light touch” (LT) and “anchor system” (AS) paradigms on postural sway. Additionally, it compared the effect of location and number of points of contact to the control of posture in young adults. The location consisted of using the anchors tied to the finger and held by the hands, and, for LT, the fingertip. For the number of points of contact, participants used two hands, and then separately the dominant hand, and the non-dominant hand, for both anchor and LT paradigms. Participants stood upright with feet-together and in tandem position while performing tasks that combined the use of anchors and LT, points of contact (hand grip and finger), and number of points of contact (two hands and one hand). In this study, the anchors consist of holding in each hand a flexible cable with the other end attached to the ground. The LT consists of slightly touching a rigid surface with the tip of the index finger. The results showed, first, that the anchors improved postural control less than did the LT. Second, they revealed that holding the anchors with the hands or with them tied to the fingertip resulted in a similar reduction in postural sway only in the tandem position. For the feet-together position, the anchors tied to the fingertip were ineffective. Similarly, the use of one or two hands did not affect the contribution of the anchors. However, using two hands in the LT condition was more effective than was one hand. Third, our results showed the presence of a temporal delay between force and center-of-pressure (COP) for the anchors, only in the AP direction with feet-together. In conclusion, overall, the anchors were less effective in reducing postural sway than was the LT. The anchors attached to fingertips were as effective as the hand-held anchors in the tandem position, yet ineffective during foot-together standing. Force-COP timing explains reduced postural sway with LT but not for the anchor; hence, exploratory and supra-postural components may be involved.

## Introduction

While performing challenging upright stance tasks, individuals can reduce postural sway with the addition of haptic cues (Mauerberg-deCastro et al., 2014). The haptic system relies on non-uniform mechanical tension that displaces an organism's tissues (Turvey and Fonseca, 2014) via active exploration to detect spatiotemporal information patterns of a (quasi-) stable or moving environment. Haptic perception combines the sensory cues from skin, joint, and muscle receptors into a single system to detect invariant aspects of the stimulus. Such an action-perception system provides information about shape, texture, motion, and forces (inertial, gravitational, and accelerative). Through haptic perception, individuals become aware of environmental contexts that arise from the body's contact with adjacent objects or surfaces (Gibson, 1966), as well from the contact of non-neural extensions (e.g., an insect's antennae, a prosthetic arm, etc.) with a similar purpose (Burton, 1993; Turvey and Carello, 2011).

Both the light touch (LT) and anchor system (AS) paradigms provide examples of how, using subtle contact, a behavioral task that relies on additional haptic information conveys stabilizing effects to a postural task (Jeka and Lackner, 1994; Mauerberg-deCastro, 2004; Baldan et al., 2014; Mauerberg-deCastro et al., 2014; Oates et al., 2017). The LT paradigm describes how an individual uses the tip of the index finger to lightly touch a rigid or non-rigid surface with minimal force level (<1 N) to reduce postural sway (Jeka and Lackner, 1994). LT provides spatial information about the body's orientation relative to the support surface (Jeka and Lackner, 1995a). This can be accomplished via cutaneous mechanoreceptors of the fingertip in contact with the touch surface, as well as via kinesthetic mechanoreceptors that detect the direction and amplitude of motion of the upper limb relative to the trunk (Holden et al., 1994). These haptic cues diminish the threshold of detection of body sway, allowing corrective actions to begin earlier than in tasks without LT (Jeka and Lackner, 1994), illustrating a feedforward mechanism.

Three pieces of evidence give support to the effectiveness of the LT for reducing postural sway. First, a biomechanical model predicts that the mechanical support caused by the LT would reduce postural sway by ~2.3%; however, the actual reduction in postural sway was ~55% (Holden et al., 1994). Second, with LT, changes in center-of-pressure (COP) position occurred ~300 ms after changes in the horizontal contact force on the touch surface (Jeka and Lackner, 1994). When participants could apply as much force as they wanted (i.e., force touch), the time delay was close to 30 ms (i.e., the two signals were practically in-phase). The 300 ms delay allows information from the LT to be used to send appropriate motor commands to muscles to adjust body position. Third, when electromyography (EMG) activity of the ankle evertor muscles (important for postural stability in the tandem position) was measured, the results pointed to a reduction of ~40 and ~70% in LT and force touch, respectively, in comparison to the condition without touch (Jeka and Lackner, 1995b). This higher EMG suggests that the leg muscles played a larger role in maintaining postural stability with LT than with the force touch (Jeka and Lackner, 1995a,b). More recently, Franzén et al. (2011) showed an increase in the axial postural tone with light touch on a rigid surface, which the investigators inferred to be a tonic control mechanism that coexists with the feedforward mechanism proposed by Jeka and Lackner (1994).

The use of non-neural extensions with flexible properties seems to provide, also, additional haptic cues (Mauerberg-deCastro, 2004; Cabe, 2011). Using the AS paradigm, researchers in various experiments have demonstrated that the anchors reduce postural sway in young adults with and without intellectual disabilities (Mauerberg-deCastro et al., 2010, 2012), adolescents with cerebral palsy (Costa, 2015) and in older adults (Moraes and Mauerberg-deCastro, 2009; Freitas et al., 2013; Silva et al., 2016). With the AS, the person holds in each hand a flexible cable with a light weight (e.g., 125 g) attached at the other end, and which rests on the ground. The individual must keep the weight, or *load*, in contact with the floor, and, at the same time, slightly pull on the cable to keep it taut. Then, while pulling, the person applies force continuously on the cable in an upward direction, which generates patterns of tension, but only enough to feel the resistance of the load that is resting (or dragging—experiments with walking) on the ground (Mauerberg-deCastro et al., 2014; Hedayat et al., 2017). Because the body sway can also alter the tension of the cables, skin receptors located on the hand—combined with information about the spatial configuration of the upper limb from the kinesthetic receptors—allow an individual to integrate a compensatory action with the anchors. Therefore, the AS task forms the basis for the haptic cues.

The AS expands the possibilities for exploration of the adjacent environment through the characteristic of *telemodality*, which means that an individual can detect the properties of a distal object using a cable-mediated connection (Cabe, 2011, 2013). Kinsella-Shaw and Turvey (1992), for example, showed that individuals could accurately perceive distance through the vibration of a taut string. Similarly, the cables of the anchors (i.e., a non-rigid tool) provide the medium necessary to convey, or *mediate*, information from an adjacent support surface to the postural control system (Mauerberg-deCastro, 2004).

Mauerberg-deCastro (2004) found that young adults, who were blindfolded and asked to balance on one foot while using the anchors, showed a reduction in postural sway, regardless of load weight (ranging from 125 to 1,000 g). A similar effect with the anchors, irrespective of the load, suggests that the reduction in postural sway does not result solely from mechanical support. Immediate adaptive AS outcomes indicate that haptic cues are instantaneously integrated into the postural control mechanism (Mauerberg-deCastro et al., 2012). However, long-term effects from the systematic use of the anchors are less straightforward (Freitas et al., 2013). Three groups of older adults practiced with the anchors in 100, 50, and 0% of trials in which they stood upright in the semi-tandem position. After 2 days of practice, only the 50% group reduced postural sway in the post-test, as compared to the pre-test, which included a baseline task performed without the anchors. This result suggests that, because of the random contrast of trials, with and without the AS, the 50% group used haptic cues that the anchors provided to recalibrate the sensory integration process and reduce postural sway.

Although several studies have pointed to a reduction in postural sway with either the LT or the AS, it is not clear, however, whether these paradigms reduce postural sway equally. This is a prominent issue because it allows us to identify if there are therapeutic advantages to using one over the other. From a practical standpoint, the possible advantage of the AS in relation to the LT paradigm lies in its portability of use (i.e., AS is a tool attached to the user) in different contexts (e.g., training or rehabilitation). Unlike the LT paradigm, which requires direct contact with a rigid and stable support surface and high technology to provide information about force level feedback, the AS is inexpensive and allows flexibility of use, including its concomitant use with dynamic tasks such as walking (Costa et al., 2015, 2018; Hedayat et al., 2017). Therefore, our first purpose was to compare the effects of the LT and AS models on the postural control of young adults.

For us to make the comparison between the paradigms more accurate, the number of contact points also needs to be manipulated, since with the LT paradigm only one contact point is commonly used, whereas with the AS paradigm, two contact points typically are used (although anchoring is experimentally possible using only one point of contact). Dickstein (2005) showed that light touch with both hands reduced postural sway more than did light touch with only one hand. It is possible that the additional number of contact points increases the influx of additional haptic information, resulting in a more effective postural response. Therefore, we tested two contact points for both the AS and the LT, as well as a single contact point for each paradigm. In this case, the dominant and non-dominant hands were tested separately, since there appears to be a difference between them concerning the two paradigms. Araújo et al. (2013) revealed that LT with the dominant hand was more beneficial in reducing postural sway than with the non-dominant hand in young adults, especially in the condition of eyes closed. Santos et al. (2015), in turn, demonstrated that the use of the AS in the non-dominant hand reduced postural sway in older adults in a way that was similar to that of using both hands, but the same did not occur with the use of the AS with the dominant hand.

Another aspect that deserves attention regarding the comparison of the anchor and LT paradigms is the point of contact with the body. With the AS, the contact of the cable across the entire palm (i.e., a grip pattern) predominates, whereas, in the light touch paradigm, contact with the tip of the index finger predominates. Mauerberg-deCastro et al. (2014) argue that the AS task context differs from LT because the former integrates haptic information from a larger degree-of-freedom mechanism in which the anchor system's point of contact couples with a relatively motion-free segmental configuration (linked hands, wrists, forearms, arms, shoulder, trunk). Still, the comparison between the paradigms relies on the manipulation of the point of contact. Thus, the AS was tested with two different points of contact: the whole hand and the tip of the finger. The fingertip region is densely populated with tactile receptors (Johansson and Vallbo, 1983), while a point of contact with a wider area of the hand poses the question of the diversity in haptic exploration strategies and the potential for congesting the information flow. The LT paradigm could be more effective in reducing postural sway due to the greater influx of information generated by the stimulation of a greater number of local tactile receptors. The use of the AS at the fingertip would make it possible to identify if the effectiveness in reducing postural sway relies on a more populated region of receptors, or if, perhaps, the hand gripping the AS, a dynamic multi-degrees-of-freedom configuration, would be more effective.

Therefore, the second purpose of the present study was to compare the effect of the number and location of the points of contact in the postural control of young adults. Such experimental manipulation would reveal how the orientation of the body is subordinate to the spatial configuration of points of surface contact (i.e., one vs. two vectors, plus the erect body, relative to adjacent surfaces), and consider the use of one or two hands.

Although it was shown that there was a time delay between the force applied on the touch bar and the displacement of the COP with the LT in young adults (Jeka and Lackner, 1994; Clapp and Wing, 1999), the existence of such a delay has not been established for the AS. A study of this relationship is necessary to identify whether the AS's pulling force is temporarily advanced relative to the displacement of the COP to allow haptic cues to be used to control the position of the body in space. Thus, the present study analyzed the phase relationship between the force measured on the anchor's cable and the displacement of the COP.

The final purpose of this study was to discriminate between the effects of task difficulty in both the LT and AS contexts. The literature about the AS paradigm reveals diverse outcomes regarding the extent of effects on postural stabilization (Moraes and Mauerberg-deCastro, 2009). Mauerberg-deCastro et al. (2010) specifically observed that when an AS postural task includes progressive demands for balance (i.e., blindfolding or increasing height of support surface), the relative benefit of reducing sway seems to be greater as the challenging context increases.

## Materials and methods

### Participants

Twenty-one healthy young adults (10 males & 11 females; 24.9 ± 2.2 years; 171.3 ± 9.3 cm; 75.1 ± 14.6 kg) participated in this study. The local Ethics Committee approved all experimental procedures.

### Procedures

Participants stood upright and with bare feet on a force plate (40 × 60 cm, Bertec, Columbus, USA), to allow for the computation of the COP, and were blindfolded. Eight cameras (MX-T40S) of the Vicon tridimensional motion capture system (Oxford, United Kingdom) tracked 39 markers, placed on specific anatomical landmarks based on the *Plug-in-Gait Full Body* model of the Vicon system, to compute the center-of-mass (COM). Signals from the force plate and the motion capture system were digitally synchronized and sampled at 200 Hz by the Nexus software of the Vicon system.

Participants performed nine experimental conditions that combined the addition of haptic information (anchors tied to fingers [AF], anchors held by the hands [AH], and LT) and the number of contact points (one hand [dominant and non-dominant] and both hands). Also, participants performed a control condition without additional haptic information (non-contact condition). Upper limb dominance was determined based on the Edinburgh Handedness Inventory (Oldfield, 1971).

**Figure 1:**
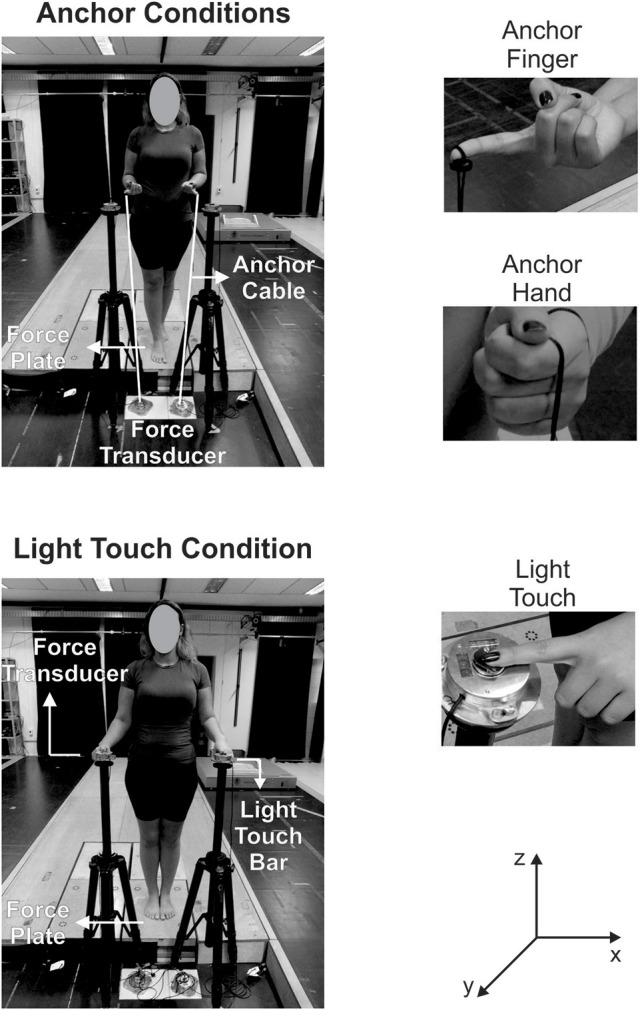
Pictures illustrating the anchor condition (only the two hands condition is shown) and the light touch condition (only the two hands condition is illustrated). For the anchor picture, a white line was superimposed on the anchors' cables to facilitate their visibility. The pictures also illustrate the position of the force plate and the force transducers to measure the force on the touch bar and the anchor cables. The pictures on the right side show how the anchor's cable was tied to the fingertip (anchor finger) or held by the participant in the hand (anchor hand) and the light touch of the fingertip on the center of the touch bar (bottom). (y: anteroposterior | x: mediolateral | z: vertical).

In the LT condition, participants touched the center (a circle with a diameter of 1.7 cm) of a flat, rigid surface with the tip of the index finger (Figure 1). We covered the surface with a wrinkled cloth to increase its texture. This textured surface was directly fixed over a 6-degrees-of-freedom force transducer (Nano17 F/T Transducer, ATI Industrial Automation, Apex, USA), with a resolution of 0.003125 N in both shear and vertical directions. We attached the force transducer to a tripod that allowed the regulation of the touch bar height. The force data (AP, ML, and vertical) from the force transducers were synchronized and digitally sampled at 200 Hz by the Nexus software, along with the force plate and the body markers. We placed the touch bar in front and to the side of the participant at a distance that allowed the surface to be touched with a comfortable arm configuration. The elbow's angle was approximately 90°. We informed the participants that the touch bar was not designed to support a large amount of force and it could not be used as a support like a cane. Although light touch studies establish a limit of 1 N of normal force on the touch bar, Tremblay et al. (2004) showed that this instruction was sufficient to maintain force levels within the limit of 1 N. When using only one hand to touch the bar, participants maintained the contralateral arm positioned at the side of the body. When using both hands, participants touched each hand (dominant and non-dominant) on a touch bar positioned on each side of their body.

In the AH condition, participants held a flexible cable, keeping the elbows at an angle of approximately 90°, tied to a force transducer (Nano17 F/T Transducer, ATI Industrial Automation, Apex, USA), which was attached to the ground (Mauerberg-deCastro et al., 2014) (Figure 1). The AS used in this study was adapted from the original AS, since the mass of 125 g was replaced by the force transducer that was fixed to the ground. The force signals were collected as explained for the LT condition. We informed participants that the fixation of the force transducer on the floor was not designed to withstand high tensile forces and could not be used as mechanical support. For the AH condition, participants held the anchor's cable in such a way that the cable looped around the palm (Figure 1). In the AF condition, the anchor's cable was tied to the tip of the index finger, with the tip of the finger pointing upwards (Figure 1). In both anchor conditions, participants were told to keep the anchor cable taut. Participants held the anchor with both hands and with only one hand (dominant or non-dominant). In the conditions with only one hand, the contralateral arm was positioned at the side of the body.

To investigate the level of difficulty of the balancing task, participants performed all experimental conditions using two foot configurations: tandem and feet-together, in separate blocks of trials counterbalanced among participants. In the tandem condition, they stood with one foot in front of the other so that the toes of the rear foot touched the heel of the fore foot. The dominant foot was placed in the front position, which was determined by asking the participants which foot they preferred to use for kicking a ball. In the feet-together condition, participants stood with feet side-by-side, with them touching each other. To ensure that participants always took the same foot position on the force plate, we drew their foot positions on it. The reduction in the size of the base of support in the medial-lateral direction in the tandem position makes this task more challenging than the feet-together position in the frontal plane. Moreover, the control of the degrees of freedom in each foot configuration is different. In the feet-together position, balance can be achieved by controlling the lower limb joints independently. In the tandem position, because the joints are highly coupled one to another, they cannot be controlled independently. The tasks become more challenging or difficult when more degrees of freedom have to be controlled.

Participants repeated three times each experimental condition, totaling 60 trials. Each trial lasted 40 s, and, between the trials, participants had a 1-min rest. They had a 5-min rest interval between the block of trials. They were informed that they could request additional rest, if needed. The order of the trials within each block was completely randomized.

### Data analysis

We filtered the COP data with a low-pass, fourth-order, Butterworth digital filter with a cut-off frequency of 5 Hz. We discarded the first and the last 5 s of each trial. The COP analysis included the calculation of the following variables: path length, mean sway speed, and the frequency at 80% of the power spectrum (F80). The path length corresponds to the distance traveled by the COP, and it was calculated as the sum of the scalar displacements of the COP between each consecutive pair of frames. For the calculation of the mean sway speed, the sway amplitude was calculated by subtracting the mean position of the COP from each point recorded in the trial. The mean sway speed was calculated as the mean of the first derivative of the sway amplitude. According to Baratto et al. (2002), F80 is the frequency measure that best discriminates the alterations of the postural control system. We conducted a spectral analysis to get the power spectrum density (PSD) of the COP displacement using Welch's method. F80 corresponds to the frequency below which it is 80% of the total power. For the calculation of F80, we computed the area under the PSD curve for later identification of the frequency corresponding to 80% of the total area. Because the more prominent body sway was in the frontal plane in the tandem position, we computed the COP variables only in the ML direction. For the feet-together position, we computed the COP variables in both AP and ML directions because the amount of body sway was equivalent in both planes.

Based on the 39 markers, the Nexus software computed the COM position. We computed the COM velocity as the first derivative of the COM position (central difference procedure) in the AP and ML directions. Afterward, we calculated the extrapolated center-of-mass (XcoM), according to the equation $XcoM=COM+\left(\frac{\dot{COM}}{\omega_{0}}\right)$ (Hof et al., 2005), where *XcoM* is the extrapolated position of the COM, *COM* is the actual position of the center-of-mass, $\dot{COM}$ is the COM velocity and $\omega_{0}=\sqrt{\frac{g}{l}}$, where *g* is the acceleration due to gravity and *l* is the height of the COM. Based on the XcoM, we computed the margin of dynamic stability (MDS) along the entire trial (see **Figure 4A** for an illustration), as follows: *MDS* = *BOS*−*XcoM*, where *BOS* corresponds to the boundary of the base of support. The definition of the BOS encompasses the markers placed on the feet (see **Figure 4A**). The 2nd metatarsal and the heel markers defined the anterior and posterior boundaries of the base of support, respectively. With feet-together, we used the average value between the two feet of the AP coordinates. The right and left boundaries were defined based on the lateral malleolus marker. The computation of the MDS relative to each boundary of the BOS took into consideration the vector direction of the COM velocity. Therefore, when the COM velocity vector was pointing toward the anterior boundary, the MDS was computed relative to that boundary. In the tandem position, we computed the MDS relative to right and left boundaries, whereas for the feet-together position we computed the MDS relative to all four boundaries. Positive value for MDS indicates that XcoM is located before the extremities of the foot, since it is dynamically stable. For statistical analysis, we used the root mean square (RMS) of the MDS relative to each direction.

The time-to-contact (TtC) was calculated over the duration of the entire trial (30 s) (**Figure 3A**), using the equation $TtC\;=\;\frac{\left(BOS-COM\right)}{\dot{COM}}$ (Van Wegen et al., 2002), where, *BOS* is the boundary of the base of support, *COM* is the current position of the center-of-mass, and $\dot{COM}$ is the velocity of the COM. After calculating the TtC of the entire time series, we used the absolute values of AP and ML TtC. Based on the work of Haddad et al. (2006), the TtC was determined by considering the various minima of the TtC time series (threshold of 40 s) (see **Figure 3A** for an example). The minima represent the transition point at which the COM reverses its direction of movement. After identifying these minima, we computed the mean TtC. For the tandem position, only the TtC in the ML direction was calculated, whereas in the feet-together position, the TtC was calculated in both AP and ML directions.

The force data were filtered by a fourth-order, low-pass, Butterworth digital filter with a 3-Hz cut-off frequency. For the vertical component, we calculated for each force transducer the average force and its variability (i.e., root mean square, RMS). Also, the temporal relationship between the force applied to the touch bar or anchors and the displacement of the COP was calculated using cross-correlation analysis. Considering that the participants in the tandem position swayed more in the frontal plane, we used the force and COP in the ML direction. In the feet-together position, however, we ran the cross-correlation in both AP and ML directions, since the COP displacement was similar in both directions. Correlations were performed forward and backward for each 0.01 s to determine if the highest correlations occurred at times other than τ = 0 (i.e., signals in-phase), assuming a maximum time delay of ±0.50 s. When using two force transducers (one for each hand), cross-correlations were performed between the force and the COP displacement for each transducer separately. In addition, to identify the relationship between forces applied by the dominant and non-dominant hands in the conditions with two hands, cross-correlation analyses were performed for each direction of the force components. The maximum correlation coefficient values and the time difference between the signals (τ) were identified.

### Statistical analysis

We used the mean values of the three trials of each experimental condition for statistical analyses. We carried out separate statistical analyses for each foot position. To identify the effect of additional haptic information on posture control (model #1) in the tandem position, we performed analyses of variance (ANOVA) for one factor (4 conditions [non-contact, AF in both hands, AH in both hands, and LT with the dominant hand]), with repeated measures for the following dependent variables: path length, mean sway speed, F80, time-to-contact, and RMS of the margin of dynamic stability. For the feet-together position, we ran a MANOVA for one factor (4 conditions), with repeated measures. The same dependent variables were analyzed, but each MANOVA combined the AP and ML directions.

In the second set of analyses (model #2), we compared the number of contact points (two hands, dominant and non-dominant hand) in all three conditions of additional haptic information (AF, AH, and LT). For the tandem condition, we performed a two-way ANOVA (3 conditions [AF, AH, and LT] x 3 contact points [two hands, dominant hand, and non-dominant hand]), with repeated measures in both factors for all dependent variables. For the feet-together position, we ran a two-way MANOVA, which included the AP and ML values of the same dependent variables described above.

For the time difference between the signals (τ) of the force and COP analysis, an ANOVA for two factors (3 conditions [AF, AH, and LT] X 4 contact points [two hands dominant, one hand dominant, two hands non-dominant, one hand non-dominant]), with repeated measures in both factors, was carried out for the tandem position. For the feet-together position, two ANOVA were performed, separately for AP and ML directions, using the same statistical model. For the cross-correlation between forces in the dominant and non-dominant hands, we ran a one-way ANOVA (3 conditions [AF, AH, and LT]), with repeated measures, for each force direction. The mean and RMS of the vertical force were analyzed by three-way ANOVA (3 conditions [AF, AH, and LT] x 2 number of contact points [one and two hands] x 2 sides [dominant and non-dominant]), with repeated measures in all factors.

*Post-hoc* tests with Bonferroni's adjustment identified the pairwise differences when main and interaction effects were identified (*p* ≤ 0.05).

## Results

The detailed results of the statistical analyses for COP variables, time-to-contact, and margin of dynamic stability are available in Tables 1–3.

**Table 1:** *F*- and *p*-values for main effect of condition of the ANOVA and MANOVA and the univariate follow-up for the postural control variables for the effect of additional haptic information (Model #1).

**Variables**	**Tandem—ANOVA**	**Feet together—MANOVA**	
Path length	*F*_(3, 60)_ = 27.743, ***p*** ≤ **0.0001**	Wilks' λ = 0.318, *F*_(6, 118)_ = 15.196, ***p*** ≤ **0.0001**	
Mean sway speed	*F*_(3, 60)_ = 27.747, ***p*** ≤ **0.0001**	Wilks' λ = 0.318, *F*_(6, 118)_ = 15.203, ***p*** ≤ **0.0001**	
F80	*F*_(3, 60)_ = 15.784, ***p*** ≤ **0.0001**	Wilks' λ = 0.290, *F*_(6, 118)_ = 16.865, ***p*** ≤ **0.0001**	
Time-to-contact	*F*_3, 60_ = 15.325, ***p*** ≤ **0.0001**	Wilks' λ = 0.433, *F*_(6, 106)_ = 9.185, ***p*** ≤ **0.0001**	
		**Follow-up univariate**	
		**AP direction**	**ML direction**
Path length	------	*F*_(3, 60)_ = 31.890, ***p*** ≤ **0.0001**	*F*_(3, 60)_ = 13.617, ***p*** ≤ **0.0001**
Mean sway speed	------	*F*_(3, 60)_ = 31.906, ***p*** ≤ **0.0001**	*F*_(3, 60)_ = 13.614, ***p*** ≤ **0.0001**
F80	------	*F*_(3, 60)_ = 22.194, ***p*** ≤ **0.0001**	*F*_(3, 60)_ = 15.009, ***p*** ≤ **0.0001**
Time-to-contact	------	*F*_(3, 54)_ = 14.573, ***p*** ≤ **0.0001**	*F*_(3, 54)_ = 8.1390, ***p*** ≤ **0.0001**
	**Tandem—ANOVA**	**Feet together—MANOVA**	
Margin of dynamic stability (RMS)	*F*_(3, 48)_ = 18.242, ***p*** ≤ **0.0001**	Wilks' λ = 0.251, *F*_(6, 112)_ = 18.558, ***p*** ≤ **0.0001**	
		**Follow-up univariate**	
Margin of dynamic stability (RMS)		**AP direction**	**ML direction**
	------	*F*_(3, 57)_ = 40.392, ***p*** ≤ **0.0001**	*F*_(3, 57)_ = 13.170, ***p*** ≤ **0.0001**

*Bold p-values indicate statistical significance. (AP: anterior-posterior | ML: medial-lateral | RMS: root mean square)*.

**Table 2:** *F*- and *p*-values for main and interaction effects (condition, number of contact points, and condition^*^number of contact points) of the ANOVA for the postural control variables for the effect of contact points and number of contact points (Model #2) in the tandem position (only medial-lateral direction).

**Variables**	**Condition**	**Number of contact points**	Condition ^*^ number of contact points
**ANOVA**			
Path length	*F*_(2, 40)_ = 101.055, ***p*** ≤ **0.0001**	*F*_(2, 40)_ = 9.342, ***p*** ≤ **0.0001**	*F*_(4, 80)_ = 5.015, ***p*** = **0.008**
Mean sway speed	*F*_(2, 40)_ = 101.101, ***p*** ≤ **0.0001**	*F*_(2, 40)_ = 9.345, ***p*** ≤ **0.0001**	*F*_(4, 80)_ = 5.020, ***p*** = **0.001**
F80	*F*_(2, 40)_ = 51.817, ***p*** ≤ **0.0001**	*F*_(2, 40)_ = 1.584, *p* = 0.218	*F*_(4, 80)_ = 4.939, ***p*** = **0.001**
Time-to-contact	*F*_(2, 36)_ = 58.700, ***p*** ≤ **0.0001**	*F*_(2, 36)_ = 13.598, ***p*** ≤ **0.0001**	*F*_(4, 72)_ = 6.897, ***p*** ≤ **0.0001**
Margin of dynamic stability (RMS)	*F*_(2, 24)_ = 61.242, ***p*** ≤ **0.0001**	*F*_(2, 24)_ = 5.005, ***p*** = **0.015**	*F*_(4, 48)_ = 5.315, ***p*** = **0.001**

*Bold p-values indicate statistical significance. (RMS: root mean square)*.

**Table 3:** *F*- and *p*-values for main and interaction effects (condition, number of contact points, and condition^*^number of contact points) of the MANOVA and the univariate follow-up for the postural control variables for the effect of contact points and number of contact points (Model #2) in the feet-together position.

**Variables**	**Condition**		**Number of contact points**		**Condition** ^*^ **Number of contact points**	
Path length	Wilks' λ = 0.10, *F*_(4, 78)_ = 43.90, ***p*** ≤ **0.0001**		Wilks' λ = 0.59, *F*_(4, 78)_ = 5.82, ***p*** ≤ **0.0001**		Wilks' λ = 0.68, *F*_(8, 158)_ = 4.16, ***p*** ≤ **0.0001**	
Mean sway speed	Wilks' λ = 0.10, *F*_(4, 78)_ = 43.92, ***p*** ≤ **0.0001**		Wilks' λ = 0.59, *F*_(4, 78)_ = 5.82, **p** ≤ **0.0001**		Wilks' λ = 0.68, *F*_(8, 158)_ = 4.16, ***p*** ≤ **0.0001**	
F80	Wilks' λ = 0.14, *F*_(4, 78)_ = 32.37, ***p*** ≤ **0.0001**		Wilks' λ = 0.31, *F*_(4, 78)_ = 15.44, ***p*** ≤ **0.0001**		Wilks' λ = 0.36, *F*_(8, 158)_ = 13.05, ***p*** ≤ **0.0001**	
Time-to-contact	Wilks' λ = 0.22, *F(*_4, 66)_ = 19.02, ***p*** ≤ **0.0001**		Wilks' λ = 0.51, *F*_(4, 66)_ = 6.63, ***p*** ≤ **0.0001**		Wilks' λ = 0.79, *F(*_8, 134)_ = 2.11, ***p*** = **0.039**	
**Follow-up univariate**	**AP direction**	**ML direction**	**AP direction**	**ML direction**	**AP direction**	**ML direction**
Path length	*F*_(2, 40)_ = 164.88, ***p*** ≤ **0.0001**	*F*_(2, 40)_ = 46.01, ***p*** ≤ **0.0001**	*F*_(2, 40)_ = 3.74, ***p*** = **0.032**	*F*_(2, 40)_ = 12.05, ***p*** ≤ **0.0001**	*F*_(4, 80)_ = 3.82, ***p*** = **0.007**	*F*_(4, 80)_ = 6.37, ***p*** ≤ **0.0001**
Mean sway speed	*F*_(2, 40)_ = 164.94, ***p*** ≤ **0.0001**	*F*_(2, 40)_ = 46.02, ***p*** ≤ **0.0001**	*F*_(2, 40)_ = 3.75, ***p*** = **0.032**	*F*_(2, 40)_ = 12.06, ***p*** ≤ **0.0001**	*F*_(4, 80)_ = 3.83, ***p*** = **0.007**	*F*_(4, 80)_ = 6.37, ***p*** ≤ **0.0001**
F80	*F*_(2, 40)_ = 115.16, ***p*** ≤ **0.0001**	*F*_(2, 40)_ = 20.81, ***p*** ≤ **0.0001**	*F*_(2, 40)_ = 36.88, ***p*** ≤ **0.0001**	*F*_(2, 40)_ = 2.34, *p* = 0.109	*F*_(4, 80)_ = 30.22, ***p*** ≤ **0.0001**	*F*_(4, 80)_ = 2.67, ***p*** = **0.038**
Time-to-contact	*F*_(2, 34)_ = 25.90, ***p*** ≤ **0.0001**	*F*_(2, 34)_ = 42.72, ***p*** ≤ **0.0001**	*F*_(2, 34)_ = 1.609, *p* = 0.215	*F*_(2, 34)_ = 15.18, ***p*** ≤ **0.0001**	*F*_(4, 68)_ = 0.757, *p* = 0.557	*F*_(4, 68)_ = 4.02, ***p*** = **0.005**
Margin of dynamic stability (RMS)	Wilks' λ = 0.088, *F*_(4, 70)_ = 41.491, ***p*** ≤ **0.0001**		Wilks' λ = 0.616, *F*_(4, 70)_ = 4.794, ***p*** = **0.002**		Wilks' λ = 0.774, *F*_(8, 142)_ = 2.432, ***p*** = **0.017**	
**Follow-up univariate**	**AP direction**	**ML direction**	**AP direction**	**ML direction**	**AP direction**	**ML direction**
Margin of dynamic stability (RMS)	*F*_(2, 36)_ = 184.45, ***p*** ≤ **0.0001**	*F*_(2, 36)_ = 32.17, ***p*** ≤ **0.0001**	*F*_(2, 36)_ = 9.01, ***p** =* **0.001**	*F*_(2, 36)_ = 0.95, *p* = 0.395	*F*_(4, 72)_ = 2.01, *p* = 0.102	*F*_(4, 72)_ = 3.97, ***p*** = **0.006**

*Bold p-values indicate statistical significance. (AP: anterior-posterior | ML: medial-lateral | RMS: root mean square)*.

### The effect of additional haptic information

#### COP

**Figure 2:**
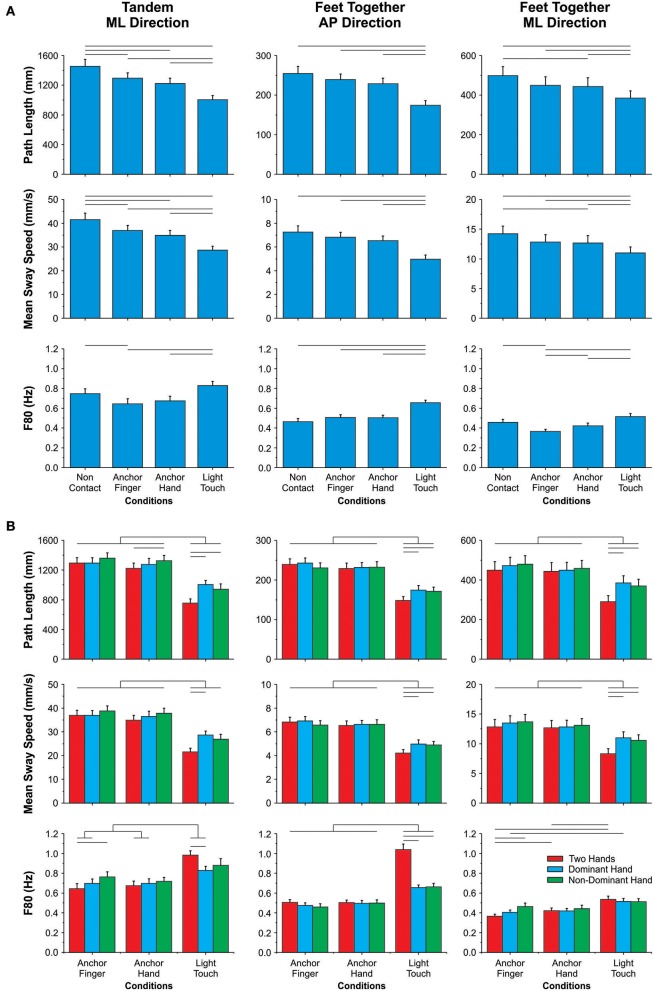
Mean and standard error of the mean for the center-of-pressure variables in both tandem (only data in the ML direction) and feet-together (data in both AP and ML directions) positions for the effect of additional haptic information **(A)** and the effect of contact points and number of contact points **(B)**. The horizontal lines indicate pairwise differences.

Figure 2A shows the results of the comparative analyses involving the non-contact condition and the AF, AH, LT conditions. For the tandem position, there was a main effect of condition in all three variables (*p* ≤ 0.0001). Path length and mean sway speed decreased gradually from the non-contact condition to the AF, AH, and LT conditions, and these conditions were also different from each other. Interestingly, F80 decreased in the AF condition as compared to both non-contact and LT conditions, and it decreased for the AH as compared to the LT condition. When standing with feet-together, MANOVA identified main effect of condition for all three variables (*p* ≤ 0.0001), and the univariate analyses indicated an effect in both AP and ML directions (*p* ≤ 0.0001). With feet-together, both path length and mean sway speed did not reduce in the AF and AH conditions as compared to the non-contact condition; however, the AH reduced both variables in the ML direction as compared to the non-contact condition. There was a decrease in path length and mean sway speed in the LT condition as compared to non-contact, AF, and AH conditions in both AP and ML directions. In the AP direction, the use of the anchors did not affect F80; however, it increased in the LT as compared to all other conditions. In the ML direction, the F80 decreased in the AF condition as compared to all other conditions, as well as decreased in the AH condition as compared to the LT condition.

#### Time-to-contact

**Figure 3:**
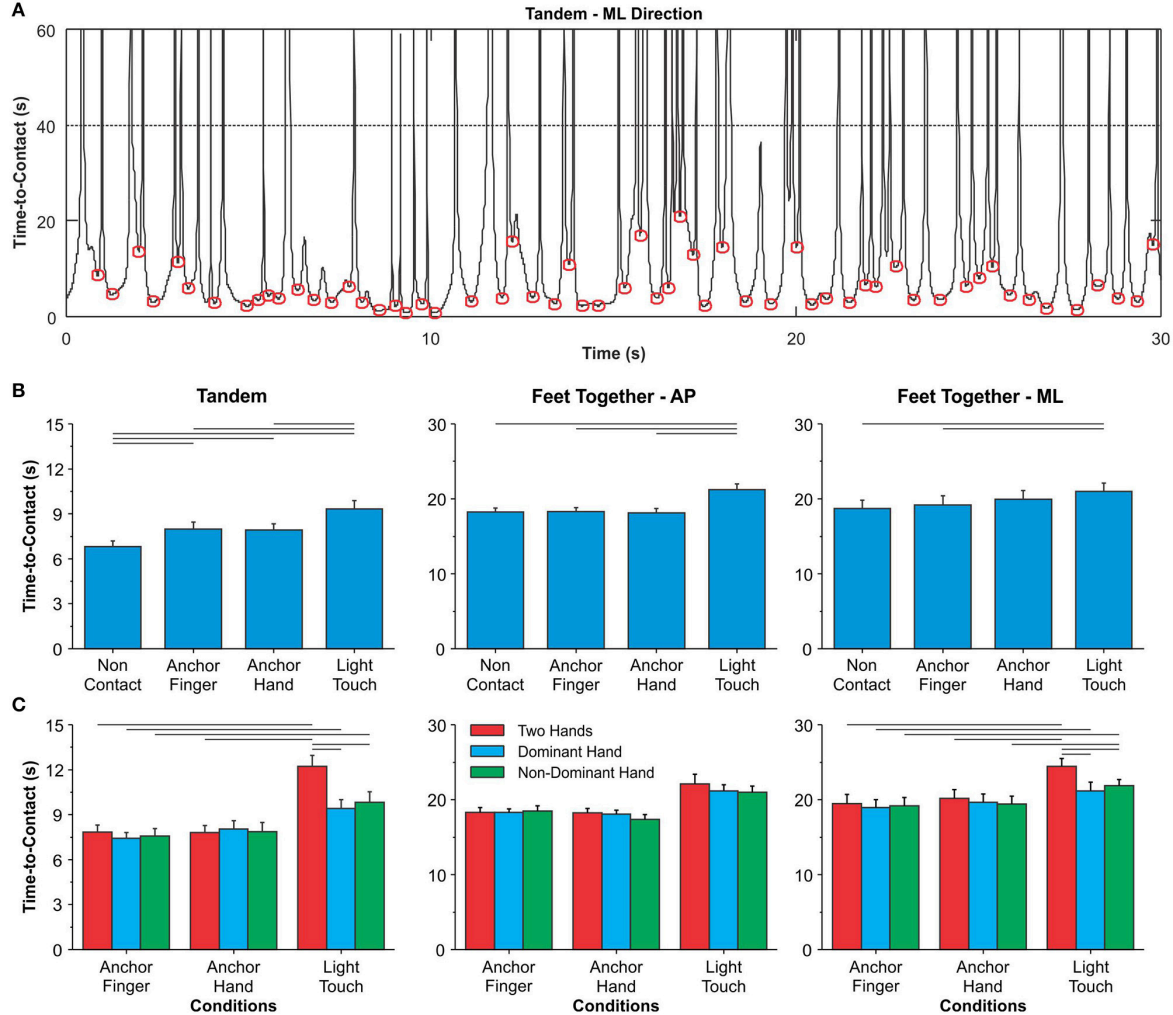
**(A)** Example of a time series for one participant for the time-to-contact. Each circle represents the several minima used to compute the mean time-to-contact. Mean and standard error of the mean for the time-to-contact in both tandem (only data in the ML direction) and feet-together (data in both AP and ML directions) positions for the effect of additional haptic information **(B)** and the effect of contact points and number of contact points **(C)**. The horizontal lines indicate pairwise differences.

Figure 3B shows the data for the non-contact conditions and the three conditions with additional haptic cues. There was a main effect of condition in the tandem position (*p* ≤ 0.0001). Both anchor conditions presented an increase in time-to-contact as compared to the non-contact condition. The time-to-contact increased in the LT condition as compared to non-contact and both anchor conditions. With feet-together, the MANOVA exhibited a main effect of condition (*p* ≤ 0.0001) in both AP and ML (*p* ≤ 0.0001) directions. The anchors did not alter the time-to-contact with feet-together; however, the time-to-contact was greater for the LT condition than for the non-contact and anchor conditions.

#### Margin of dynamic stability

**Figure 4:**
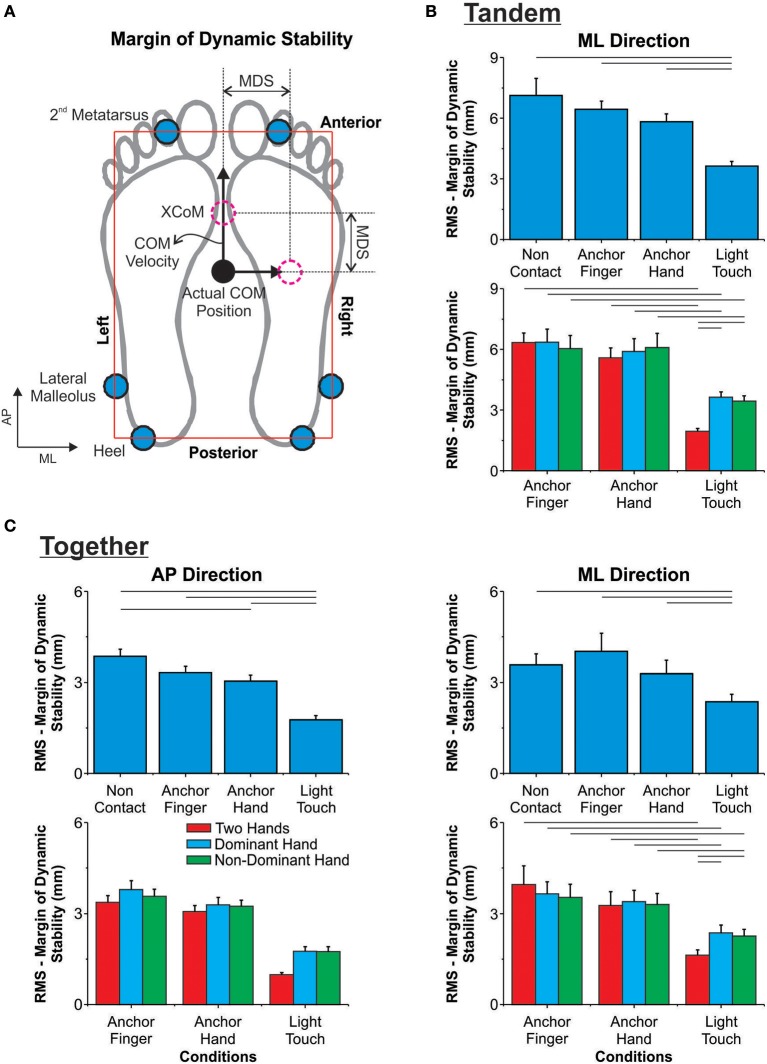
**(A)** Schematic representation of the extrapolated center-of-mass (XcoM), the boundaries of the base of support, and the computation of the margin of dynamic stability (MDS). **(B)** Mean and standard error of the mean for the root mean square (RMS) of the margin of dynamic stability in the tandem position (only ML direction) for the effect of additional haptic information (top row) and the effect of contact points and number of contact points (bottom row). **(C)** Mean and standard error of the mean for the root mean square (RMS) of the margin of dynamic stability in the feet-together position (AP and ML directions) for the effect of additional haptic information (top row) and the effect of contact points and number of contact points (bottom row). The horizontal lines indicate pairwise differences.

For the tandem position, the ANOVA identified a main effect of condition (*p* ≤ 0.0001). The anchors did not affect the RMS of the margin of dynamic stability; however, the LT reduced it as compared to the non-contact condition in the tandem condition (Figure 4B). With feet-together, there was a main effect of condition in the MANOVA (*p* ≤ 0.0001), followed by univariate tests showing the effect in both directions (*p* ≤ 0.0001). The AH condition reduced the RMS as compared to the non-contact condition in the AP direction. The LT condition reduced the RMS in both directions (Figure 4C).

### The effect of contact points and number of contact points

#### COP data

Figure 2B shows the results of the comparative analyses involving only the additional haptic information conditions and the number of contact points. For the tandem position, there was an interaction between conditions and number of contact points for all three dependent variables (*p* ≤ 0.008). There was no difference between AF and AH for the different contact points. The use of both hands in the AH condition resulted in reduced path length as compared to the AH condition with only the non-dominant hand. Path length and mean sway speed reduced in the LT condition as compared to the AF and AH conditions for all number of contact points, and the use of two hands revealed an even greater reduction in the values for these variables in the LT condition. In the AF condition, there was an increase in the F80 only when the non-dominant hand was used as compared to both hands. The F80 showed an increase in the LT condition, particularly with the use of both hands. In the feet-together condition, the MANOVA identified an interaction between conditions and number of contact points for all three dependent variables (*p* ≤ 0.0001), and the univariate analyses identified the interaction effect in both AP and ML directions (*p* ≤ 0.04). There was no difference between AF and AH in any of the number of contact points for path length and mean sway speed. The values of these two variables reduced in the LT condition as compared to the AF and AH conditions for all number of contact points, and the use of two hands even further reduced the values of these variables in the LT condition. In the AP direction, there was no difference between the anchor conditions for the F80; however, in the ML direction, the F80 increased when the non-dominant hand was used as compared to both hands in the AF condition. In the LT condition, F80 increased in all number of contact points and in both directions, but particularly with the use of two hands.

#### Time-to-contact

Figure 3C shows the results of the analyses involving the haptic conditions and the number of contact points. In the tandem position, there was an interaction between condition and number of contact points (*p* ≤ 0.0001). Time-to-contact was not different between anchor conditions. It was longer for the LT conditions than for the AF conditions, for all number of contact points. LT with two hands resulted in longer time-to-contact than LT with one hand and AH with two hands. In the feet-together position, the MANOVA identified main effects of condition (*p* ≤ 0.0001) and number of contact points (*p* ≤ 0.0001), and interaction between these two factors (*p* = 0.039). The univariate test showed the effect of condition in both AP and ML directions (*p* ≤ 0.0001), whereas the main effect of number of contact points and the interaction was present only in the ML direction (*p* ≤ 0.005). Time-to-contact for the AF and AH conditions was smaller than for the LT condition in both AP and ML directions. In the ML direction, time-to-contact was the longest in the LT condition with the use of two hands.

#### Margin of dynamic stability

The ANOVA for the tandem position identified an interaction between conditions and number of contact points (*p* = 0.001). As illustrated in Figure 4B, the AF and AH were not different from each other in either of the contact point conditions. The RMS reduced for the LT condition irrespective of the number of contact points as compared to the AF and AH conditions. The use of two hands in the LT condition reduced the RMS even more than did the use of only the dominant or non-dominant hand. With feet-together, the MANOVA identified an interaction between conditions and number of contact points (*p* = 0.017), and the univariate test showed the interaction only in the ML direction (*p* = 0.006). As shown in Figure 4C, the anchor conditions differed from each other in all contact point conditions. The LT condition reduced the RMS as compared to both AF and AH for all number of contact points in the ML direction. Additionally, the use of the LT with two hands reduced the RMS even more as compared to that of one contact point. The MANOVA also identified main effects of condition (*p* ≤ 0.0001) and number of contact points (*p* = 0.002), with the univariate tests showing these effects in the AP direction (*p* ≤ 0.001). For the condition effect, the RMS reduced from AF to AH (*p* = 0.006) and from AH to LT (*p* ≤ 0.0001). For the effect of the number of contact points, the RMS was smaller for the use of two hands than for one.

### Force applied on the cables of the anchors and on the rigid surface

**Figure 5:**
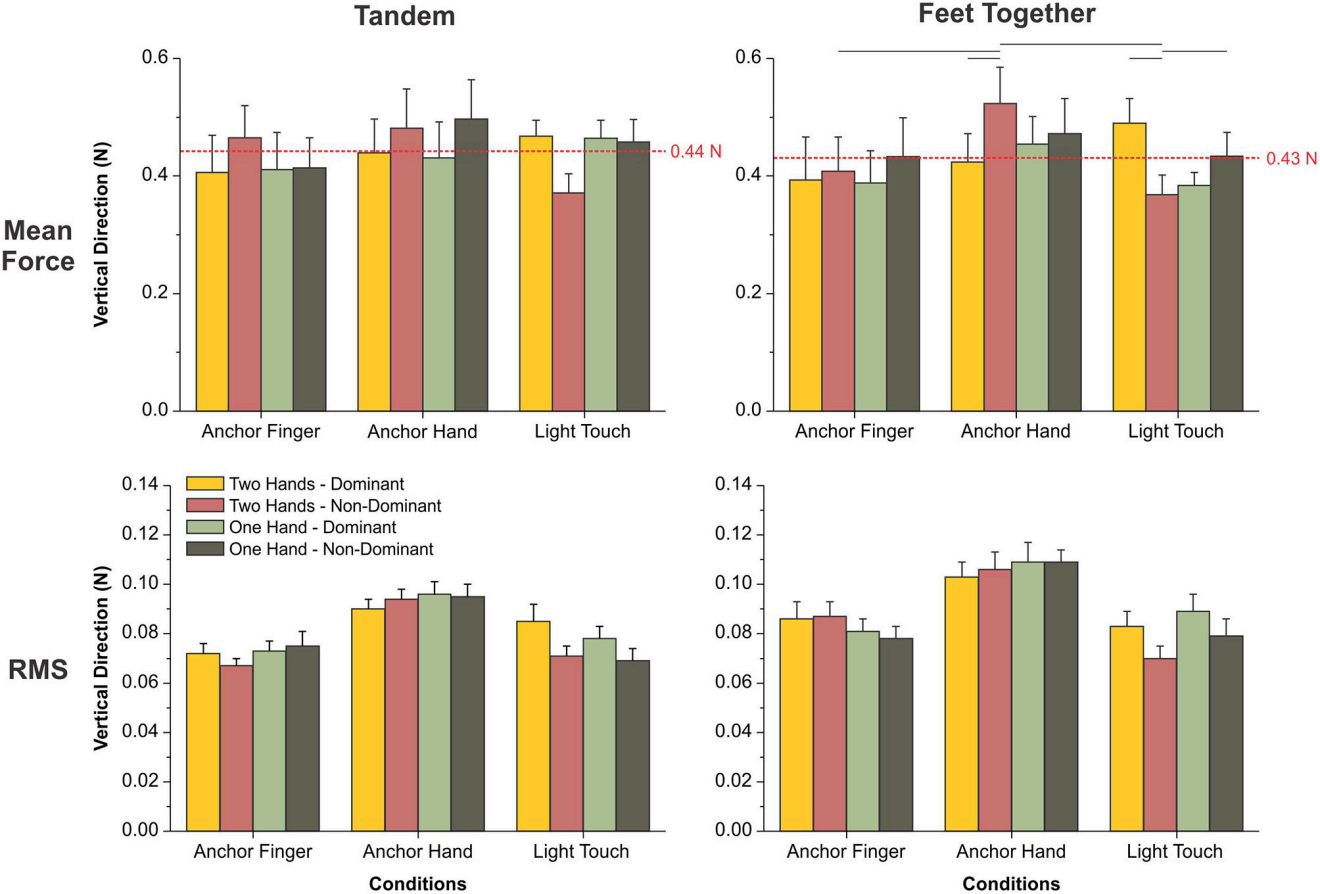
Mean and standard error of the mean for the mean vertical force (top row) and the root mean square of the vertical force (RMS, bottom row) in the tandem (left column) and feet-together (right column) positions. The horizontal lines indicate pairwise differences.

In the tandem position, there was no main or interaction effect. The mean force across different conditions was equal to 0.44 N (±0.04 N) (Figure 5). For the RMS, there was a main effect of condition [*F*_(2, 40)_ = 14.640, *p* ≤ 0.0001] and interactions between conditions and number of contact points [*F*_(2, 40)_ = 3.847, *p* = 0.030] and between conditions and sides [*F*_(2, 40)_ = 4.728, *p* = 0.014]. The RMS was larger for the AH than for the AF and LT conditions (Figure 5). For the LT condition, the RMS was larger during the use of one hand vs. two, and for the dominant hand as compared to the non-dominant hand.

With feet-together, there was an interaction between conditions, number of contact points and sides [*F*_(2, 40)_ = 6.442, *p* = 0.004) for the mean force. The mean force was higher for the AH than for the AF and LT in the non-dominant hand during the use of two hands (Figure 5). It was also larger for the non-dominant than for the dominant side in the AH during the use of two hands. For the LT, the mean force was higher for the dominant than for the non-dominant side during the use of two hands. It was larger for the non-dominant side during the use of one hand vs. two. For the RMS, there was a main effect of condition [*F*_(2, 40)_ = 16.924, *p* ≤ 0.0001] and an interaction between condition and side [*F*_(2, 40)_ = 5.039, *p* = 0.011]. The RMS was larger for the AH condition than for the AF and LT conditions. For the LT condition, the RMS was larger for the dominant than for the non-dominant side.

### The temporal relationship between COP and force

**Figure 6:**
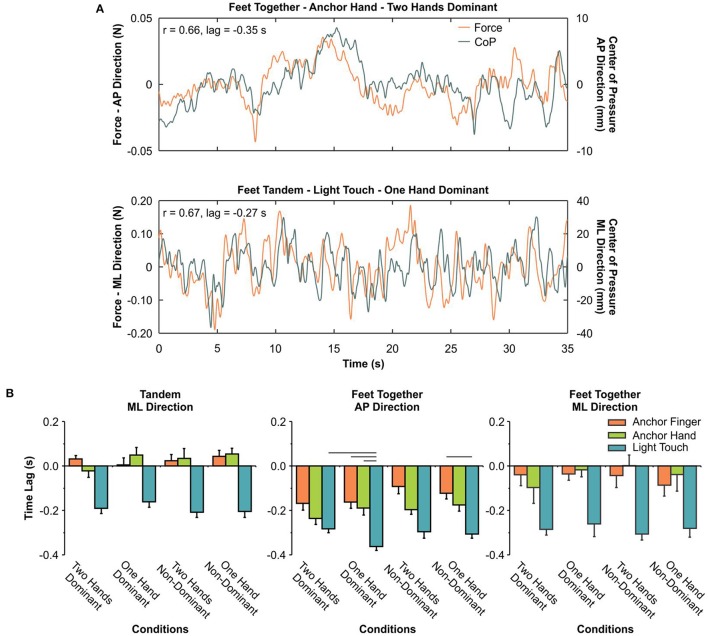
**(A)** Example of a time series for one participant for the force applied on the cable of the anchor (top) and on the touch bar (bottom), together with the displacement of the center-of-pressure (CoP). The results of the cross-correlation are shown for each of these exemplary time series. **(B)** Mean and standard error of the mean for the time lag of the cross-correlation analyses. The horizontal lines indicate pairwise differences.

Figure 6A shows overlaid time series of the COP and force relationship with a good correlation coefficient and a negative time lag between these signals, indicating that changes in COP occurred after changes in force. Interestingly, not all participants in all conditions exhibited a peak within the ±0.50-s time window. The percentage of participants who showed a peak varied from 71–100, 76–100, and 67–91% for AH, AF and LT, respectively. In Figure 6B, we see the group result for the time lag. In the tandem position, there was a main effect for conditions [*F*_(2, 26)_ = 43.580, *p* ≤ 0.0001]. For both AF and AH conditions, the time lag was close to zero and different from the LT condition (*p* ≤ 0.0001). For the feet-together condition in the AP direction, there were main effects for conditions [*F*_(2, 26)_ = 19.123, *p* ≤ 0.0001] and an interaction between conditions and contact points [*F*_(6, 78)_ = 2.575, *p* = 0.025]. The AF produced a smaller time lag than AH and LT. The time lag was smaller in the AH than in the LT. For the interaction, the differences between conditions are shown in Figure 6B. For the feet-together condition in the ML direction, there was a main effect for conditions [*F*_(2, 14)_ = 39.334, *p* ≤ 0.0001]. Time lag was close to zero in both AF and AH conditions, with values smaller than in the LT condition.

Overall, for the AF and AH conditions, the time lag was close to zero, both in feet-tandem and feet-together in the ML direction (0.02 and −0.02 s on average, respectively), indicating that changes in COP and force occurred approximately at the same time. Only in the feet-together condition in the AP direction, did AF and AH exhibit a time lag larger than zero (−0.13 and −0.20 s, respectively). For the LT condition, there always was a time lag (−0.27 s on average), indicating that changes in COP occurred after changes in the force applied to the rigid surface. It is important to note, however, that not all participants presented a significant correlation within the time window of ±0.50 s. On average, 87.2% of the participants in the different conditions tested exhibited a significant correlation, which means that some participants improved their postural control parameters without showing a temporal relationship between COP and force applied to the cables or to the touch surface.

### The temporal relationship between dominant and non-dominant force

In the tandem position, the time relationship between the dominant and non-dominant sides was close to zero for the forces in all three directions (AP: 0.00 ± 0.01 s | ML: −0.01 ± 0.02 s | Vertical: 0.00 ± 0.00 s), and the different conditions did not influence it. With feet-together, the temporal relationship was also close to zero in all directions (AP: −0.02 ± 0.01 s | ML: −0.02 ± 0.02 s | Vertical: 0.00 ± 0.00 s), and the different conditions did not influence it.

## Discussion

Our first aim was to compare the effect of the AS and LT paradigms on the postural control of young adults. In both paradigms, as expected, postural sway was reduced when compared to the non-contact condition. The results showed that the anchors improved postural control less than did the LT. Our second aim was to compare the effect of the number and location of the points of contact during the control of posture. The results showed that holding the anchors with the hands, or having the anchors attached to the fingertip resulted in similar reductions in postural sway only in the tandem position. For the feet-together position, the anchors attached to the fingertips were ineffective. Similarly, the use of one or two hands did not affect the contribution of the anchors. However, the use of two hands in the LT condition was more effective than was one hand. Our third purpose was to analyze the phase relationship between the force measured on the anchor's cable and the displacement of the center-of-pressure. Our results showed the presence of a temporal delay between force and COP for the anchors only in the AP direction, with feet-together. The fourth manipulation involved the level of difficulty of the balancing task (i.e., tandem and feet-together). The results, as highlighted above, were not similar for these two postures. The anchors attached to the fingertip were ineffective in reducing postural sway in the feet-together position; a reduction in postural sway occurred only when the task became more difficult, such as in the feet-tandem position. Holding the anchors with the hands and LT were effective in reducing body sway in both balancing tasks. These findings are discussed in detail in the following sections.

The instructions given to the participants were enough for them to avoid using the rigid bar or the cables as mechanical support, as Tremblay et al. (2004) also demonstrated. The mean force was close to 0.4 N, below the threshold typically used in light touch studies (<1 N). This is important because an elevated level of forces is indicative of increased mechanical support (Holden et al., 1994). Thus, we are confident that our participants did not use the touch bar or the anchors' cables to support their weight.

### The anchors improved postural control less than did the LT

Although the interpretation of the meaning of the postural variables used in the present study may vary according to the context and task performed in different studies (Van Emmerik and Van Wegen, 2002), the changes we observed indicate that the addition of haptic cues contributes to better postural control. This influx of sensory information helps the control system improve its references to the body's orientation relative to the ground/contact surface. This, in turn, minimizes the extent of excursions of the COP that near a *toppling* point in exploratory actions (Cabe and Pittenger, 1992; Carpenter et al., 2010). In this scenario, the reduction in COP and the limits of dynamic stability variables, together with the increase in time-to-contact, indicate an improvement in postural control. The increased time-to-contact, in all conditions, indicates that, to enhance body stability, the COM reverted its movement direction relative to the border of the base of support earlier—with these additional haptic cues—than it would have without them. In addition, the variability of the margin of dynamic stability reduced in the presence of the additional haptic cues. This result also suggests an improvement in postural control, since the distance between the extrapolated center-of-mass and the boundaries of the base of support were less variable.

Why were the anchors less effective in reducing postural sway than the LT? Haptic anchoring, with a similar role in providing aid to the orientation function of the body, which uses subtle exploratory strategies (the light maneuvering of a tool or lightly touching a surface), presents a distinct paradigmatic configuration in the haptic information-gathering process. The anchor paradigm incorporates a great number of degrees-of-freedom in its tasks (e.g., upward pulling movements, movements in the anterior-posterior and medial-lateral directions, and varied possibilities of rotation of the hand/cable around a fixed contact on the ground), which expands redundancy for the exploratory system (Mauerberg-deCastro et al., 2014). Too, the characteristics of the anchor system's non-rigid, or flexible, portion (i.e., the cable) couples with numerous joints and segments, which then converge and propagate mechanical tension that arises between these biological and non-biological “extensions.” The increased force variability that we observed when the anchor cables were held in the hand seems to support this explanation. Conversely, the LT haptic process evolves from an interplay between a fixed, fine-tuned area of the body (i.e., the finger) and a restricted, stable surface. In this case, a limited number of degrees-of-freedom of movements (i.e., vertical and horizontal forces only) are embedded in the postural task. As compared to the LT task, the gathering of haptic information through a non-rigid tool such as the anchoring system is distinctively unique. The lessor reduction in postural sway we found in the AS, compared to the LT, as well as other findings, suggest that each paradigm might be task-context dependent. For example, a recent study shows that when participants walked, LT was not as effective to enhance balance control as was the AS (Hedayat et al., 2017). These authors found that, during their walking task, the anchor system was more effective in reducing trunk velocity in the frontal plane than was LT. This result can be interpreted as a function of the type of task to be controlled.

These contrasting results with our present study, which uses standing postural tasks, also might be explained by the notion of degrees-of-freedom. When individuals walk while dragging the anchors over the floor, the behavioral system is forced to reduce the number of degrees-of-freedom. With the LT paradigm, lightly touching a handrail while walking imposes the need to precisely control the amount of force applied to the railing, and, consequently, the configuration of the arm to maintain the finger slightly over the railing. In this scenario, the number of degrees-of-freedom to be controlled increase, since there is the need to control the vertical, AP and ML position of the finger in space. Occurring simultaneously, is a need for the body to compensate for its trunk movement to keep the finger accurately in space, which, in this case, may reduce the effectiveness of this haptic tool.

Another aspect to consider regarding the uniqueness of each of these paradigms is that the anchors did not increase postural sway frequency, whereas it increased in the LT condition during the upright stance. This, combined with a reduction in the magnitude of postural sway, shows that LT seems to increase body stiffness. In fact, Franzén et al. (2011) found an increase in axial postural tone with LT on a rigid surface, which potentially increases body stiffness. They argued in favor of a tonic control mechanism that coexists with the feedforward mechanism proposed by Jeka and Lackner (1994).

### The effects of holding the anchors with the hand or attached to the fingertip had equivalent impact on postural sway only in the tandem position

The benefits of the anchors attached to the fingertip or held in the hands were similar in the tandem position as compared to the non-contact condition. However, for the feet-together condition, differences from the non-contact condition were observed only for the AH in the ML direction. As pointed out by Mauerberg-deCastro et al. (2014), the effectiveness of the anchor system is dependent on the postural demand or level of difficulty. The tandem position creates a high demand for postural control in the ML direction, which allows both anchor conditions to influence and reduce postural sway. In the feet-together position, the amount of postural sway is much greater in the ML than in the AP direction, which helps to explain the effect of the AH in reducing postural sway only in the ML direction. However, it does not explain the absence of effect of the AF in the same condition. Perhaps, because a feet-together postural task does not prompt a large amount of sway, additional and precise haptic cues from an AF task are either inaccurate or dismissed. Because the contact surface with the AF is reduced, the individual may have more difficulty in discriminating between changes in cable tension due to postural sway and hand movements. To enhance the effectiveness of the haptic cues, it seems necessary to have a greater discrepancy between these two factors on the anchors' cable tension.

The full potential of the anchors seems to be dependent on the number of receptors it stimulates, as well as on the movement synergy derived from a combination of intrinsic patterns of force tension that propagates across limb segments and throughout the multi-scale levels of tissues and beyond the organism's boundaries, which ultimately produces a single behavioral outcome (Mauerberg-deCastro et al., 2014; Turvey and Fonseca, 2014). Silva et al. (2016) showed in older adults that fixing the anchors to the forearms resulted in similar reductions in postural sway as holding the anchors in the hands. Although the highest density of tactile mechanoreceptors is in the fingertips (Johansson and Vallbo, 1983), the benefits of anchors attached to the hand vs. the digits are likely due to the dynamic activity of the hand-finger structures involved in the handling of the anchor system. Such dynamics, although subjected to variant forces over time, transform the surrounding landscape to keep information invariant or constant. The perceptual constancy concept (Gibson, 1979; Michaels and Carello, 1981; Pagano et al., 1993) justifies how the haptic sense detects the physical properties of an object or a surrounding environment, and, in this case, no matter where the point of anchor contact is, an invariant net of information proves to be efficient enough to carry out the task at hand.

### The use of one or two hands did not affect the contribution of the anchors to postural control in young adults

In the anchor conditions, the use of two hands did not result in consistent changes in postural sway, except for the reduction in path length when two hands were compared to the non-dominant hand in the AH condition. This result contradicts a prior study that showed that the use of the anchors in the non-dominant hand reduced postural sway in older adults in a similar way as using both hands, but the same did not occur with the use of the anchor system with the dominant hand (Santos et al., 2015). However, since the result in the present study was observed in only one variable in the tandem position, this observation may be related to chance. A consistent finding in the present study, however, is the absence of the additive effect of two hands with the anchors. The temporal relationship, which was close to zero between dominant and non-dominant hands, indicates that the force modulation on these sides was in-phase for both the anchor and LT conditions. Therefore, a coupling between dominant and non-dominant sides was not the reason for the different results regarding postural sway.

The difference in degrees-of-freedom between the anchor and the LT may explain why the anchors were not dependent on unilateral or bilateral cues. As explained above, when compared to the LT, the higher amount of sway in the AS is likely due to the organism's difficulty in discriminating the haptic information resulting from body sway from the activity of the cable tension. Furthermore, the in-phase coupling between dominant and non-dominant sides implies that both hands and the fingers detected the same small amount of haptic information because of the larger number of degrees-of-freedom in the anchoring task. Although the dominant and non-dominant sides were also in-phase for the LT condition, the reduced number of degrees-of-freedom present in the direct contact with a surface facilitated the detection of extra information with the two hands, explaining the reduction in postural sway.

Our results for the LT condition, with two vs. one hand, agree with the findings of Dickstein (2005), who demonstrated that LT with both hands reduced the postural sway more than did LT with only one hand. This finding is consistent, even when using different variables to capture postural sway. According to Dickstein, there is an additive effect of the haptic cues obtained by both hands, which increases the sensory influx to the postural control system.

### Changes in force preceded changes in COP in the AP direction only, for the anchors with feet-together

For the LT condition, there always was a characteristic temporal delay between the force applied to the touch bar and the displacement of the COP of ~0.27 s. Notably, this delay was independent of the foot position, and it appeared in both AP and ML directions with feet-together. This delay means that the changes in force preceded changes in the COP. This result is consistent with other studies that showed delays of 0.35 s (Clapp and Wing, 1999) and 0.29 s (Jeka and Lackner, 1994). This delay indicates a feedforward mechanism that controls posture with the addition of haptic cues (Jeka and Lackner, 1994; Franzén et al., 2011). During the delay, there is a processing of the afferent information obtained through LT, the transmission of the appropriate motor commands to the muscles, and, consequently, a reduction in postural sway. However, as Franzén et al. (2011) noted, this proposition explains only the changes in the phasic postural muscle activity. It does not explain the potential tonic changes in postural control with LT. The fact that not all participants in all conditions showed consistent time delay supports the need to understand other mechanisms that would explain postural sway reduction. Franzén et al. (2011) showed an overall increase in axial postural tone activity, which helps explain the striking and consistent finding that LT reduces postural sway. These researchers argue that this increase in axial postural tone results from a change in the reference system: from a “foot-in-space” without light touch to a “trunk-in-space” with light touch. In the former, the individuals interpret the rotation of the trunk over a stable base formed by the feet in contact with the ground; whereas, in the latter, because their hand contacts the bar, they changed their frame of reference.

The results for the anchors were not consistent with regard to the directions of the mechanisms that control posture (i.e., lack of temporal-phasic relationship in AS). In fact, the time delay was observed only in the AP direction for the feet-together position. Perhaps more importantly, the time delay was longer for the AH (~0.20 s) than for the AF (~0.13 s). These results suggest that other mechanisms may be involved in postural control enhancement through additional haptic cues provided by the anchors. The time delay of the AF condition was not enough to produce any improvement in postural control, as was observed for all the variables we analyzed in the AP direction, in the feet-together condition. On the other hand, the AF condition was sufficient to reduce postural sway in the tandem position, without any time delay between force and COP. For the AH condition, the time delay observed in the AP direction with feet-together can be related to a reduction in the margin of dynamic stability variability in the anterior and posterior boundaries of the base of support. One possibility is the exploratory component of the anchors, particularly with the hands. Evidence of the exploratory mechanism is the increase in variability of the force applied to the cables. This exploratory mechanism would allow for a better representation of the body's orientation relative to the ground, which then would allow for the sending of more accurate motor commands to the muscles. Another possible explanation for some of the benefits of the anchors relative to sway reduction is the supra-postural component of this task. According to Riley et al. (1999), posture can be investigated as “an end in itself” (p. 796) or as a response to the demands of another task (e.g., touching, looking, object manipulation, reading, and others). In the former, it is implied that the sensory inputs are used fundamentally to control stance and the main goal is to maintain postural stability. In the latter, posture is controlled with reference to other tasks or behavioral goals that might be performed when standing, which are called supra-postural tasks (Riley et al., 1999). Thus, when handling the anchors, the postural control system is subordinated to the supra-postural task, which is, in this case, to keep the cables taut without applying too much force. As noted by Riley et al. (1999), postural sway reduction with LT may be a result of posture facilitation to execute the precision task of slightly touching a small surface. In the case of the AF, the demand to keep the cable taut and stable in space using the fingertip may characterize a supra-postural task. The reduced amount of force variability in the AF condition as compared to the AH condition reinforces this argument.

### Limitation

A possible limitation of the present study is the use of adapted anchors (attached to string gauge cells). As explained in the introduction, the original anchors are comprised of a cable, which is attached to a small bag containing a slight load (typically 125 g). However, since the force applied to the cables was relatively light (0.4 N), we are confident that the adapted anchors accurately duplicated the properties of the original ones.

## Conclusions

The anchor system, as compared to the light touch, had a smaller effect on the stabilization of posture. Anchors attached to the fingertip did not improve the efficacy of the anchor system tool. Thus, the anchor held by the hand is preferable. The effects of holding the anchors with the hand or attached to the fingertip had equivalent impact on postural sway only for the most difficult posture (i.e., tandem position). The anchors tied to the fingertip were ineffective to reduce postural sway in the feet-together position. The number of contact points affected only the light touch condition, and the use of two hands resulted in less postural sway than the use of only one hand (dominant or non-dominant). Finally, the temporal delay between imprinted touch force and COP does not explain the improved postural control with the use of the anchors. It seems that the exploratory component of the anchors via the hands or the supra-postural component of the anchors via the fingertip also are involved in reducing postural sway. Both the light touch and the anchor system paradigms hold unique dynamic contexts in which degrees-of-freedom possibilities prompt positive outcomes in stabilizing body posture. The amount of postural sway reduction does not reflect a hierarchy between the two approaches. Rather, it reflects the desired outcomes of individual task constraint processes in the action-perception cycle. Each paradigm shows a task-context dependency, where unique mechanisms of the task design prompt a collective motor synergy influenced by intention (e.g., supra-postural capability) affecting the extent of exploratory strategies. The role haptic information plays on posture control mechanisms of such dual cooperative tasks varies according to their distinctive task designs (e.g., tandem vs. feet-together positions). The AS does convey aspects of LT in its task structure such as the subtle haptic contact, but AS also has unique aspects (e.g., availability of a larger number of degrees-of-freedom, indirect mediated contact, direction of applied forces) that create potential redundancy, and, yet, information for adaptive solutions.

Our long-term goal is to determine the effectiveness of using additional haptic cues in rehabilitation environments for individuals with balance problems. Our results showed that the anchors were effective in reducing body sway contingent upon the level of difficulty of the standing tasks, but, overall, LT showed a better outcome. These facts may determine what paradigm would be more suitable as a method for stimulating the postural control system. It is important to note that during dynamic tasks such as walking, the anchors were more effective in improving balance control (Hedayat et al., 2017). Thus, a combination of these two tools, depending on the nature of the task, could potentially be more effective than using only one of them in both static and dynamic tasks. Future studies should combine these two haptic tools in a rehabilitation context to identify the best combination to improve balance control.

## Ethics statement

This study was carried out in accordance with the recommendations of the National Commission for Research Ethics of the Ministry of Health, Brazil. The Ethics Committee of Human Research of the School of Physical Education and Sport of Ribeirão Preto, University of São Paulo, approved the present study. All subjects gave written informed consent in accordance with the Declaration of Helsinki.

## Author contributions

RM and EM-dC conceived and designed the experiment. BB, LS, and RB performed the experiment. RM, BB, LS, RB, and PS analyzed data. RM, BB, LS, RB, PS, and EM-dC wrote and edited the manuscript. All authors gave final approval for publication.

### Conflict of interest statement

The authors declare that the research was conducted in the absence of any commercial or financial relationships that could be construed as a potential conflict of interest.
